# Supplementary material for: Limited progress in nutrient pollution in the U.S. caused by spatially persistent nutrient sources
Source: PLoS One. 2021 Nov 29;16(11):e0258952. doi: 10.1371/journal.pone.0258952 (PMC8629290; doi:10.1371/journal.pone.0258952)
Supplement: S1 Fig — Boxplots show the interquartile range and median (middle horizontal line) and notches show the 95% confidence interval around the median. (DOCX) [file pone.0258952.s001.docx]

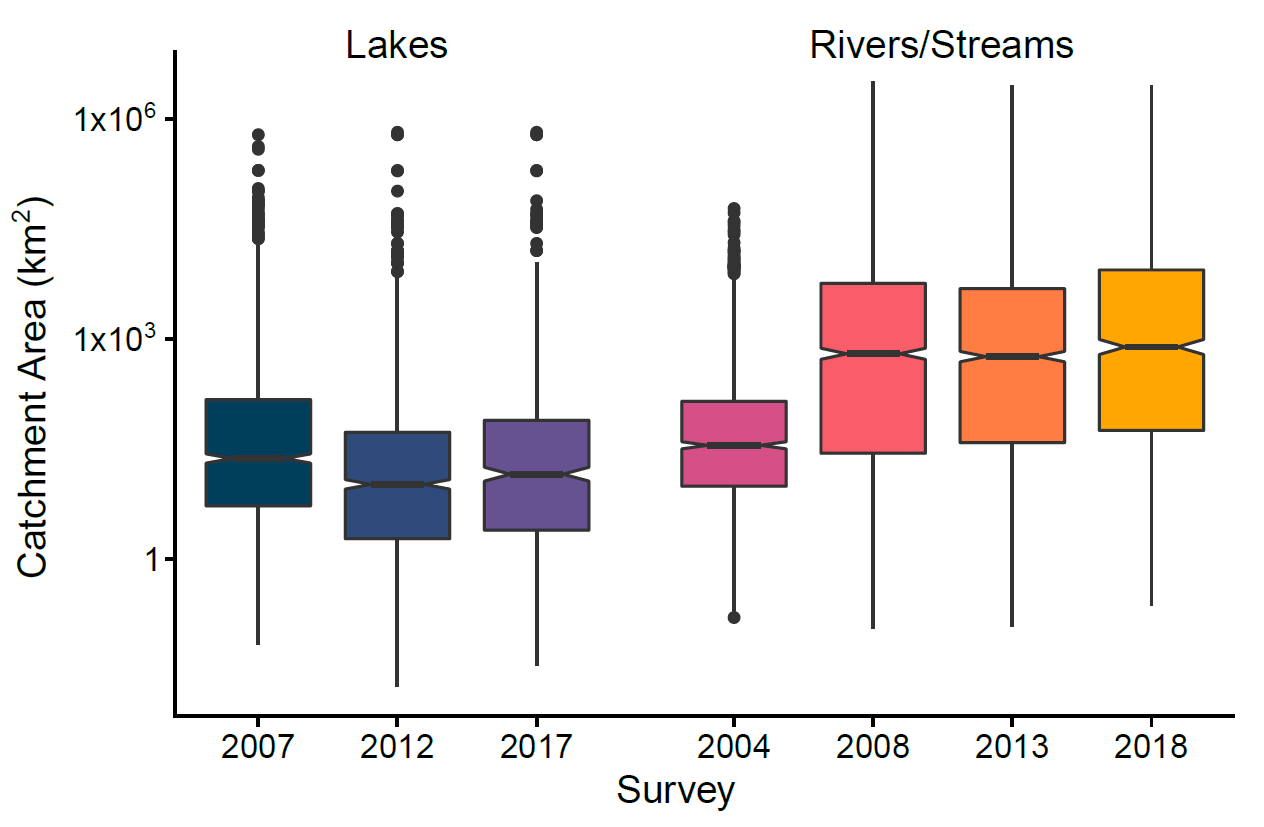


**Fig S1.** Variability of catchment sizes among the National Aquatic Resource Surveys. Boxplots show the interquartile range and median (middle horizontal line) and notches show the 95% confidence interval around the median.
