# Supplementary material for: Limited progress in nutrient pollution in the U.S. caused by spatially persistent nutrient sources
Source: PLoS One. 2021 Nov 29;16(11):e0258952. doi: 10.1371/journal.pone.0258952 (PMC8629290; doi:10.1371/journal.pone.0258952)
Supplement: S2 Fig — The red line (y = √0.5) delineates where at least half of the spatial pattern is preserved across samplings. (DOCX) [file pone.0258952.s002.docx]

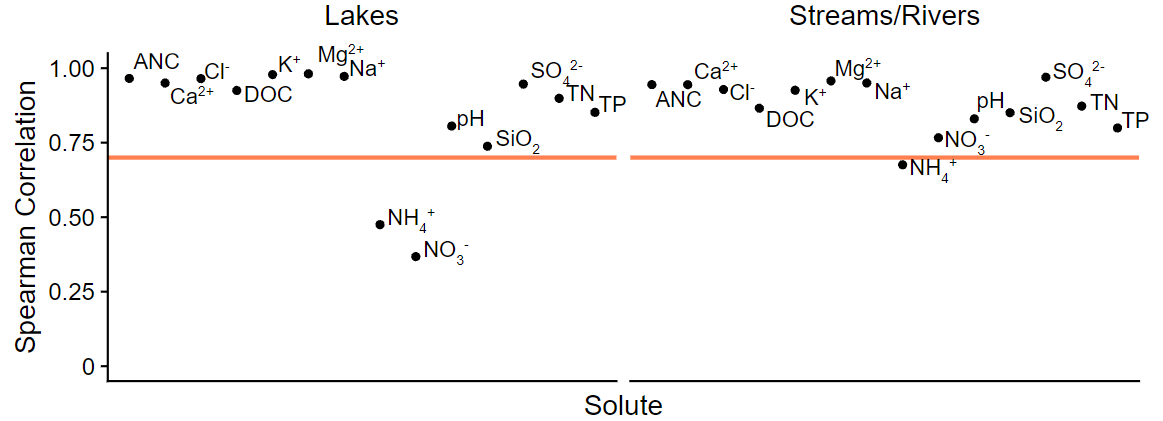


**Fig S2.** Spatial persistence at the national level for all major nutrients and ions. The red line (y = √0.5) delineates where at least half of the spatial pattern is preserved across samplings.
