# Supplementary figures and images for: Limited progress in nutrient pollution in the U.S. caused by spatially persistent nutrient sources
Source: PLoS One. 2021 Nov 29;16(11):e0258952. doi: 10.1371/journal.pone.0258952 (PMC8629290; doi:10.1371/journal.pone.0258952)

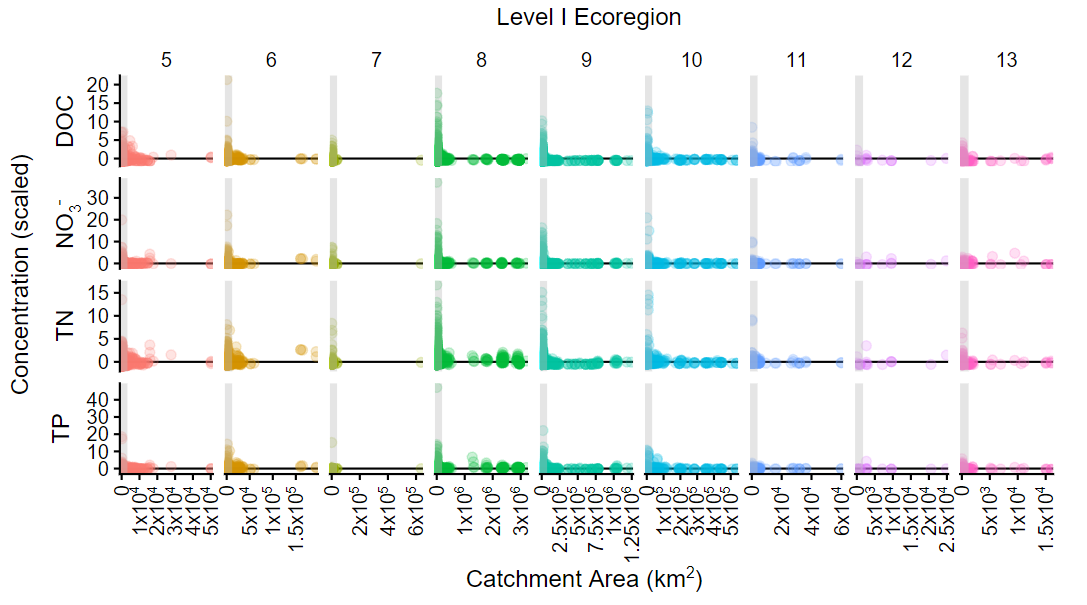


**Fig S3.** Variance collapse thresholds determined by PELT analysis for each ecoregion.

Supplement: S3 Fig — (DOCX) [file pone.0258952.s003.docx]

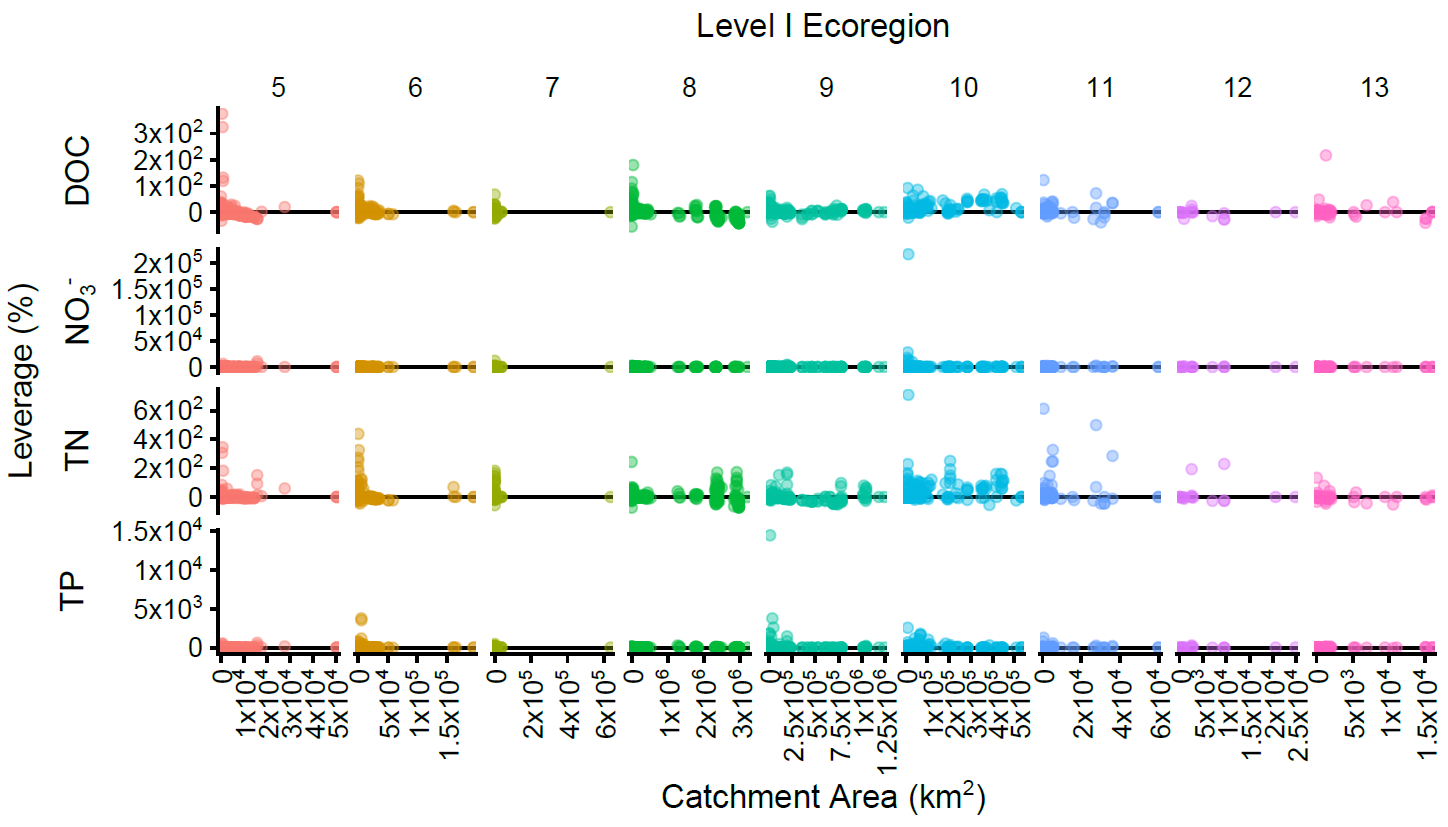


**Fig S6.** Subcatchment leverage by ecoregion.

Supplement: S6 Fig — (DOCX) [file pone.0258952.s006.docx]
