# Supplementary material for: Limited progress in nutrient pollution in the U.S. caused by spatially persistent nutrient sources
Source: PLoS One. 2021 Nov 29;16(11):e0258952. doi: 10.1371/journal.pone.0258952 (PMC8629290; doi:10.1371/journal.pone.0258952)
Supplement: S4 Fig — Boxplots show the interquartile range and median (middle horizontal line) of discharge data. Notches show the 95% confidence interval around the median. (DOCX) [file pone.0258952.s004.docx]

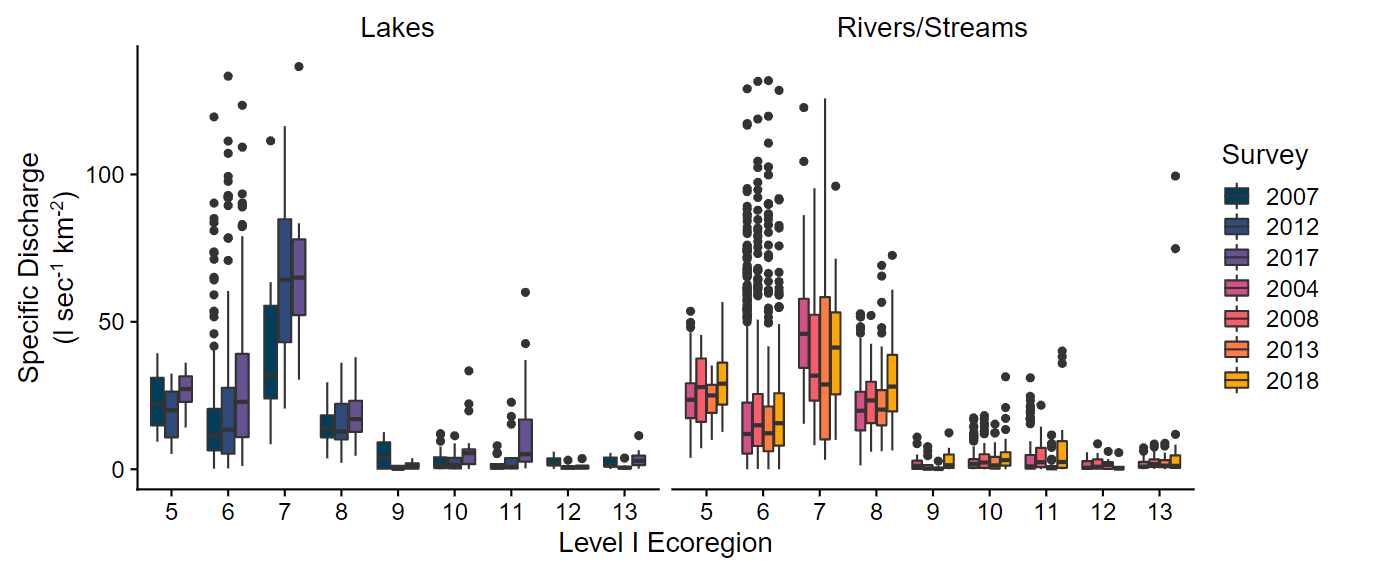


**Fig S4.** Variability of specific discharge at neighboring USGS reference stations for the EPA National Aquatic Resource Surveys by ecoregion. Boxplots show the interquartile range and median (middle horizontal line) of discharge data. Notches show the 95% confidence interval around the median.
