# Supplementary material for: Limited progress in nutrient pollution in the U.S. caused by spatially persistent nutrient sources
Source: PLoS One. 2021 Nov 29;16(11):e0258952. doi: 10.1371/journal.pone.0258952 (PMC8629290; doi:10.1371/journal.pone.0258952)
Supplement: S5 Fig — The horizontal gray line marks where the 250 km2 patch size lies in relation to stream order. Stream orders ≤ 3 are considered headwater streams. (DOCX) [file pone.0258952.s005.docx]

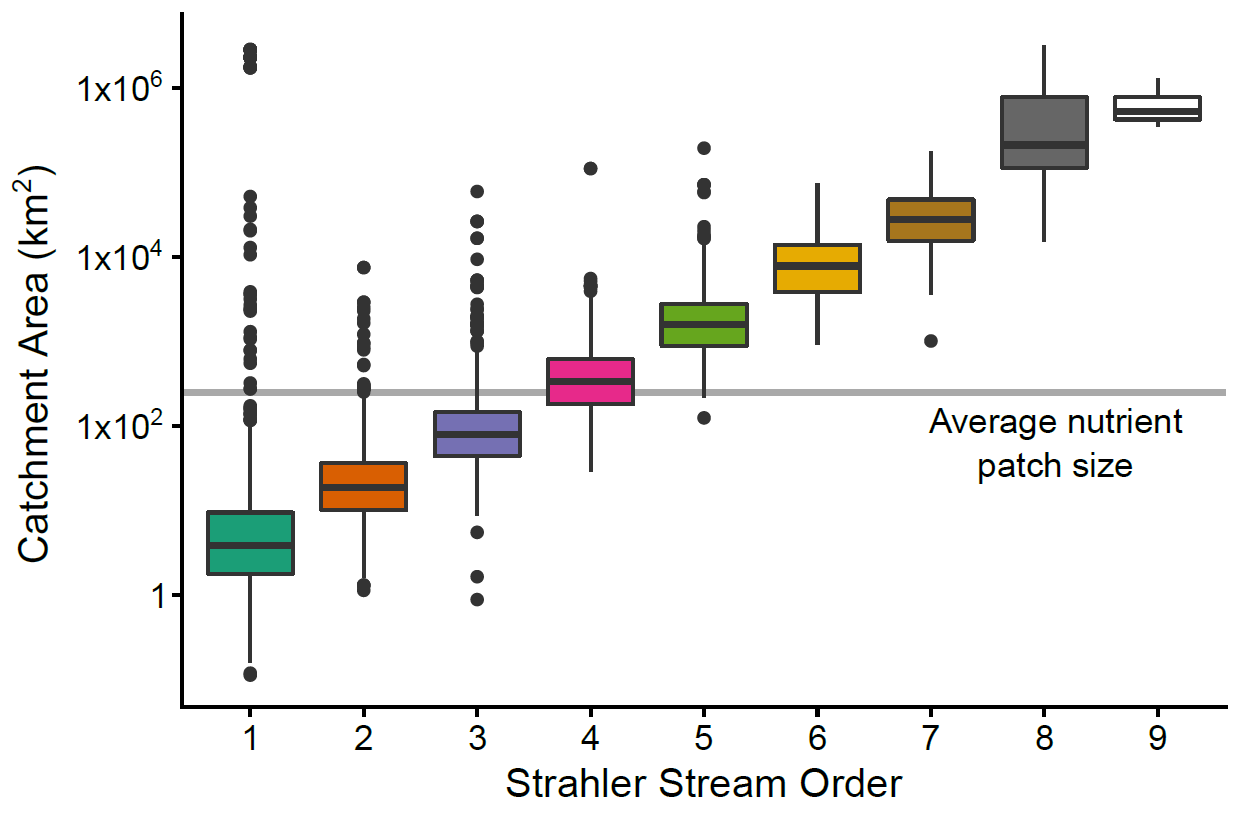


**Fig S5.** Boxplots of Strahler stream order and catchment area. The horizontal gray line marks where the 250 km^2^ patch size lies in relation to stream order. Stream orders ≤ 3 are considered headwater streams.
