# Supplementary material for: Limited progress in nutrient pollution in the U.S. caused by spatially persistent nutrient sources
Source: PLoS One. 2021 Nov 29;16(11):e0258952. doi: 10.1371/journal.pone.0258952 (PMC8629290; doi:10.1371/journal.pone.0258952)
Supplement: S7 Fig — Models predicted nutrient concentration (plot A) and subcatchment leverage (an estimate of nutrient flux; plot B) using catchment characteristics and climate variables from the EPA National Aquatic Resource Surveys and WorldClim database. (DOCX) [file pone.0258952.s007.docx]

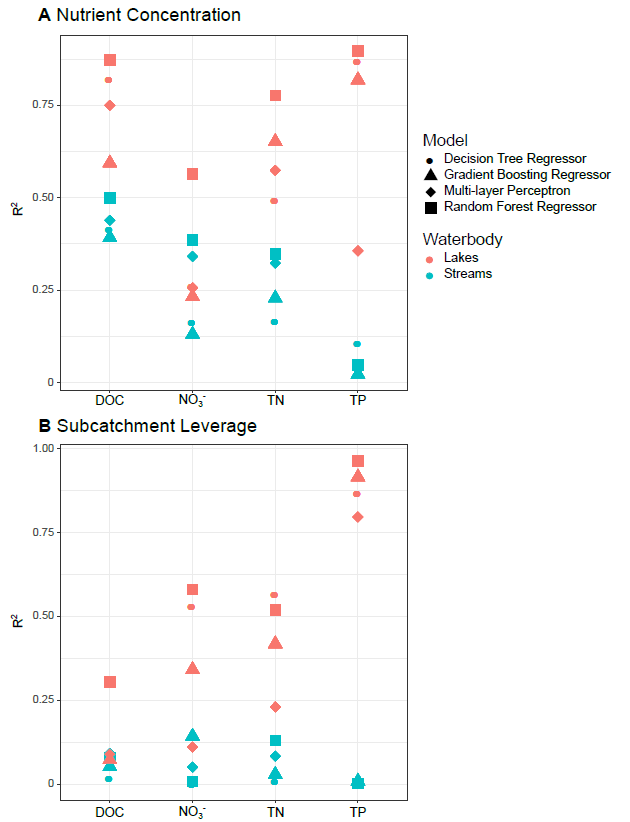


**Fig S7.** R-squared values for machine learning models. Models predicted nutrient concentration (plot A) and subcatchment leverage (an estimate of nutrient flux; plot B) using catchment characteristics and climate variables from the EPA National Aquatic Resource Surveys and WorldClim database.
