# Supplementary material for: Limited progress in nutrient pollution in the U.S. caused by spatially persistent nutrient sources
Source: PLoS One. 2021 Nov 29;16(11):e0258952. doi: 10.1371/journal.pone.0258952 (PMC8629290; doi:10.1371/journal.pone.0258952)
Supplement: S8 Fig — Bi-plots between lake and stream model predictor importance show the most important predictors among the models predicting subcatchment leverage. (DOCX) [file pone.0258952.s008.docx]

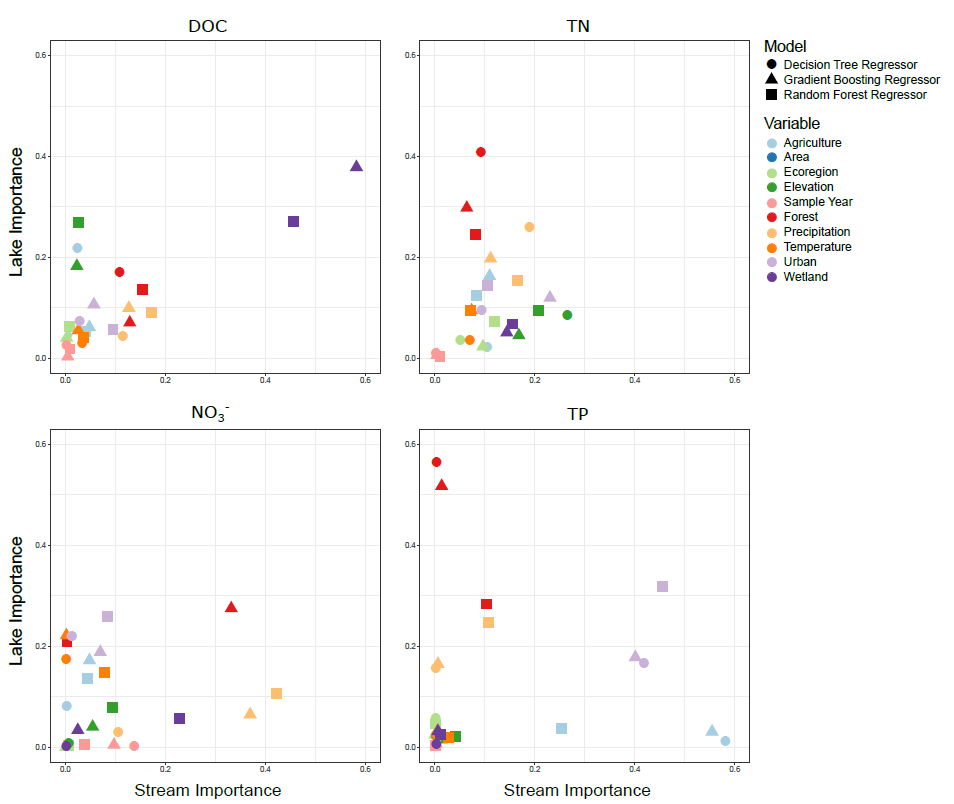


**Fig S8.** Machine learning models illustrating the relationship between catchment characteristics, climate variables, and subcatchment leverage (an estimate of nutrient flux). Bi-plots between lake and stream model predictor importance show the most important predictors among the models predicting subcatchment leverage.
