# Supplementary material for: Limited progress in nutrient pollution in the U.S. caused by spatially persistent nutrient sources
Source: PLoS One. 2021 Nov 29;16(11):e0258952. doi: 10.1371/journal.pone.0258952 (PMC8629290; doi:10.1371/journal.pone.0258952)
Supplement: S9 Fig — They show how the predictions partially depend on the input variables of interest and are able to depict whether the relationship is linear, curvilinear, or a step function. The plots above show the partial dependence plots for the major predictors of DOC (A), NO3- (B), TN (C), and TP (D) subcatchment leverage for lakes and streams. (DOCX) [file pone.0258952.s009.docx]

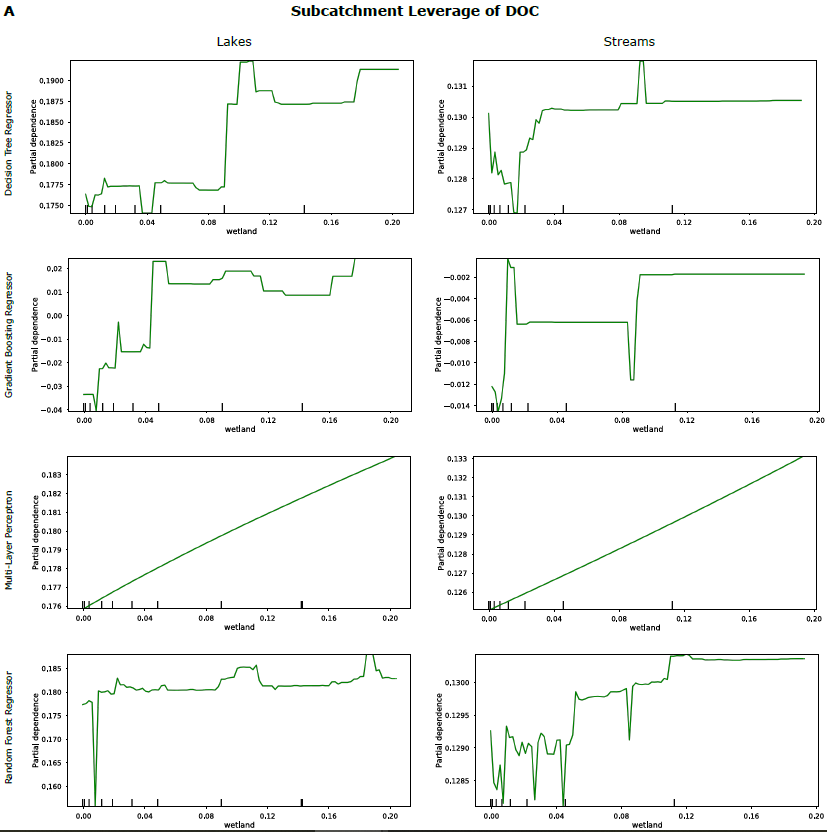


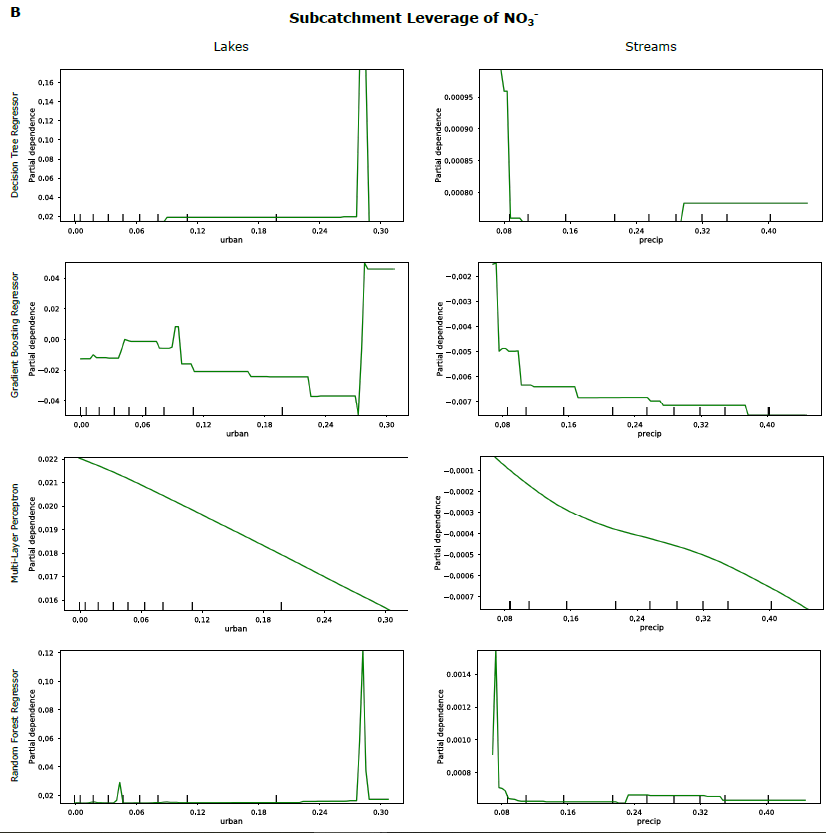


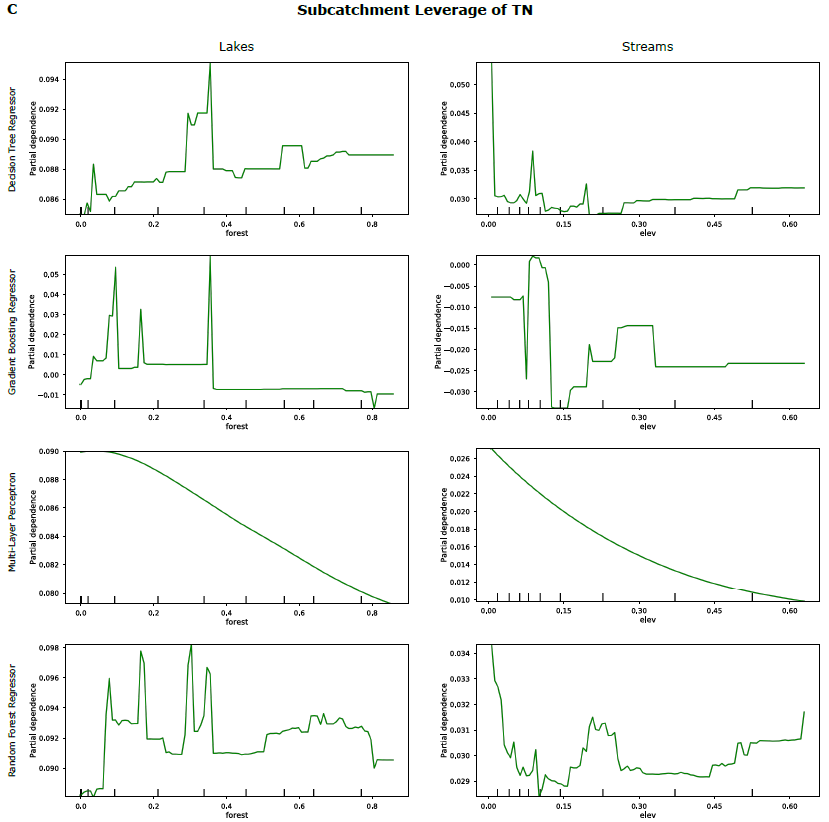


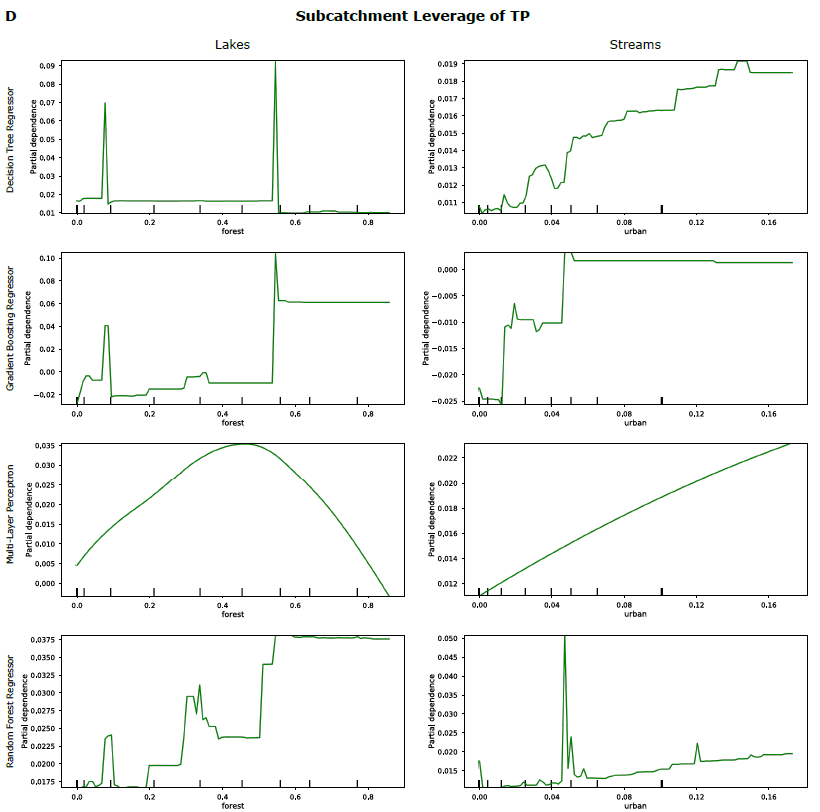


**Fig S9.** Partial dependence plots show the modeled relationship between subcatchment leverage (an estimate of nutrient flux) and the primary predictors for lakes and streams. They show how the predictions partially depend on the input variables of interest and are able to depict whether the relationship is linear, curvilinear, or a step function. The plots above show the partial dependence plots for the major predictors of DOC (A), NO_3_^-^ (B), TN (C), and TP (D) subcatchment leverage for lakes and streams.
