# Supplementary material for: Limited progress in nutrient pollution in the U.S. caused by spatially persistent nutrient sources
Source: PLoS One. 2021 Nov 29;16(11):e0258952. doi: 10.1371/journal.pone.0258952 (PMC8629290; doi:10.1371/journal.pone.0258952)
Supplement: S1 Table — (DOCX) [file pone.0258952.s010.docx]

| **Table S1. Summary of the number of sites in each EPA National Aquatic Resource Survey (NARS).** | | | | | | |
| --- | --- | --- | --- | --- | --- | --- |
| Assessment | Year(s) | Abbreviation | Water body type | Total water chemistry samples | Unique sites | Overlapping sites with previous survey |
| National Lakes Assessment (NLA) | 2007 | NLA 07 | Lakes | 1326 | 1141 | NA |
| National Lakes Assessment (NLA) | 2012 | NLA 12 | Lakes | 1230 | 1127 | 406 |
| National Lakes Assessment (NLA) | 2017 | NLA 17 | Lakes | 1210 | 1099 | 495 |
| Wadeable Streams Assessment (WSA) | 2000-2004 | WSA 04 | Rivers/Streams | 1392 | 1391 | NA |
| National Rivers and Streams Assessment (NRSA) | 2008-2009 | NRSA 08 | Rivers/Streams | 2320 | 2114 | 357 |
| National Rivers and Streams Assessment (NRSA) | 2013-2014 | NRSA 13 | Rivers/Streams | 2261 | 1985 | 790 |
| National Rivers and Streams Assessment (NRSA) | 2018-2019 | NRSA 19 | Rivers/Streams | 2112 | 1914 | 931 |
