# Supplementary material for: Limited progress in nutrient pollution in the U.S. caused by spatially persistent nutrient sources
Source: PLoS One. 2021 Nov 29;16(11):e0258952. doi: 10.1371/journal.pone.0258952 (PMC8629290; doi:10.1371/journal.pone.0258952)
Supplement: S2 Table — (DOCX) [file pone.0258952.s011.docx]

| **Table S2. Nutrient summary statistics per ecoregion.** | | | | | | | | | | | |
| --- | --- | --- | --- | --- | --- | --- | --- | --- | --- | --- | --- |
|  | | | | DOC (mg L^-1^) | | NO_3_^-^-N (mg L^-1^) | | TN (mg L^-1^) | | TP (μg L^-1^) | |
| Ecoregion | Waterbody | Survey | n | mean | σ | mean | σ | mean | σ | mean | σ |
| 5 – Northern Forests | Lakes | NLA 07 | 137 | 6.34 | 4.11 | 0.01 | 0.04 | 0.46 | 0.35 | 15.80 | 25.40 |
|  |  | NLA 12 | 138 | 7.14 | 4.95 | 0.01 | 0.05 | 0.50 | 0.28 | 23.93 | 18.74 |
|  |  | NLA 17 | 160 | 7.74 | 4.84 | 0.00 | 0.01 | 0.48 | 0.24 | 20.44 | 18.40 |
|  | Rivers/Streams | WSA 04 | 67 | 7.18 | 6.27 | 0.56 | 1.06 | 0.47 | 0.31 | 24.58 | 34.00 |
|  |  | NRSA 08 | 178 | 8.19 | 8.91 | 0.11 | 0.22 | 0.55 | 0.56 | 44.72 | 113.79 |
|  |  | NRSA 13 | 175 | 8.94 | 11.45 | 0.14 | 0.28 | 0.55 | 0.45 | 40.23 | 39.98 |
|  |  | NRSA 18 | 183 | 7.01 | 7.74 | 0.19 | 0.42 | 0.46 | 0.45 | 18.11 | 45.74 |
| 6 – Northwestern Forested Mountains | Lakes | NLA 07 | 150 | 3.61 | 6.19 | 0.02 | 0.05 | 0.28 | 0.25 | 27.74 | 74.47 |
|  |  | NLA 12 | 162 | 3.68 | 4.14 | 0.01 | 0.03 | 0.34 | 0.38 | 38.33 | 40.47 |
|  |  | NLA 17 | 144 | 4.00 | 5.68 | 0.01 | 0.02 | 0.42 | 0.57 | 31.31 | 53.22 |
|  | Rivers/Streams | WSA 04 | 418 | 1.56 | 1.13 | 0.22 | 0.49 | 0.16 | 0.20 | 23.79 | 46.55 |
|  |  | NRSA 08 | 186 | 1.85 | 1.75 | 0.04 | 0.12 | 0.19 | 0.27 | 45.33 | 56.75 |
|  |  | NRSA 13 | 261 | 1.87 | 1.61 | 0.04 | 0.10 | 0.17 | 0.18 | 52.93 | 67.51 |
|  |  | NRSA 18 | 194 | 1.94 | 1.96 | 0.04 | 0.08 | 0.16 | 0.15 | 23.18 | 22.73 |
| 7 – Marine West Coast Forests | Lakes | NLA 07 | 29 | 4.58 | 3.06 | 0.03 | 0.04 | 0.51 | 0.49 | 28.34 | 42.49 |
|  |  | NLA 12 | 23 | 4.11 | 3.27 | 0.17 | 0.53 | 0.50 | 0.63 | 50.13 | 53.17 |
|  |  | NLA 17 | 32 | 5.46 | 3.73 | 0.01 | 0.03 | 0.60 | 0.60 | 47.02 | 51.50 |
|  | Rivers/Streams | WSA 04 | 43 | 1.71 | 1.10 | 0.66 | 0.89 | 0.24 | 0.22 | 18.86 | 18.93 |
|  |  | NRSA 08 | 40 | 1.32 | 1.18 | 0.09 | 0.15 | 0.40 | 1.04 | 301.52 | 1667.41 |
|  |  | NRSA 13 | 45 | 1.36 | 1.06 | 0.19 | 0.75 | 0.29 | 0.78 | 69.01 | 110.74 |
|  |  | NRSA 18 | 24 | 1.20 | 0.83 | 0.25 | 0.90 | 0.34 | 0.96 | 23.24 | 16.01 |
| 8 – Eastern Temperate Forests | Lakes | NLA 07 | 538 | 5.93 | 3.84 | 0.10 | 0.42 | 0.89 | 1.33 | 58.03 | 114.49 |
|  |  | NLA 12 | 497 | 6.35 | 5.53 | 0.11 | 0.60 | 0.95 | 1.13 | 81.22 | 201.97 |
|  |  | NLA 17 | 475 | 6.13 | 3.70 | 0.14 | 0.52 | 0.94 | 0.90 | 67.73 | 103.75 |
|  | Rivers/Streams | WSA 04 | 377 | 3.95 | 3.94 | 4.47 | 11.29 | 1.41 | 2.71 | 84.56 | 195.51 |
|  |  | NRSA 08 | 1116 | 5.00 | 7.09 | 0.81 | 1.80 | 1.38 | 2.17 | 126.02 | 384.90 |
|  |  | NRSA 13 | 971 | 5.15 | 5.28 | 0.98 | 2.07 | 1.50 | 2.22 | 118.36 | 196.17 |
|  |  | NRSA 18 | 991 | 4.56 | 4.61 | 1.08 | 1.93 | 1.29 | 1.80 | 47.64 | 93.26 |
| 9 – Great Plains | Lakes | NLA 07 | 351 | 15.58 | 17.06 | 0.07 | 0.19 | 2.35 | 3.00 | 234.86 | 334.90 |
|  |  | NLA 12 | 283 | 13.31 | 11.03 | 0.23 | 3.08 | 2.14 | 3.56 | 244.72 | 395.49 |
|  |  | NLA 17 | 284 | 14.14 | 14.10 | 0.17 | 1.87 | 2.14 | 2.76 | 224.16 | 379.93 |
|  | Rivers/Streams | WSA 04 | 239 | 7.13 | 5.44 | 8.18 | 19.89 | 2.94 | 5.09 | 258.91 | 553.30 |
|  |  | NRSA 08 | 533 | 5.88 | 5.51 | 0.94 | 3.13 | 1.74 | 3.35 | 278.67 | 744.33 |
|  |  | NRSA 13 | 503 | 6.92 | 5.91 | 1.17 | 3.37 | 2.10 | 3.67 | 268.59 | 538.47 |
|  |  | NRSA 18 | 482 | 6.44 | 5.12 | 1.16 | 2.57 | 1.76 | 2.66 | 164.62 | 366.81 |
| 10 – North American Deserts | Lakes | NLA 07 | 88 | 7.27 | 7.49 | 0.10 | 0.29 | 0.95 | 1.08 | 244.57 | 767.46 |
|  |  | NLA 12 | 86 | 6.00 | 5.52 | 0.03 | 0.09 | 0.84 | 1.07 | 136.73 | 187.79 |
|  |  | NLA 17 | 73 | 7.17 | 8.85 | 0.10 | 0.23 | 0.85 | 0.71 | 90.53 | 145.56 |
|  | Rivers/Streams | WSA 04 | 141 | 2.49 | 1.90 | 1.43 | 4.80 | 0.63 | 1.12 | 79.65 | 221.26 |
|  |  | NRSA 08 | 210 | 3.06 | 2.13 | 0.22 | 0.73 | 0.72 | 2.20 | 151.57 | 453.98 |
|  |  | NRSA 13 | 176 | 3.20 | 2.92 | 0.28 | 0.72 | 0.78 | 2.07 | 203.26 | 501.74 |
|  |  | NRSA 18 | 187 | 4.03 | 7.71 | 0.23 | 0.60 | 0.51 | 0.79 | 90.56 | 418.65 |
| 11 – Mediterranean California | Lakes | NLA 07 | 12 | 4.39 | 3.81 | 0.02 | 0.06 | 0.75 | 1.03 | 214.83 | 418.81 |
|  |  | NLA 12 | 22 | 5.76 | 5.81 | 0.04 | 0.11 | 0.52 | 0.55 | 75.98 | 164.07 |
|  |  | NLA 17 | 17 | 5.64 | 3.60 | 0.16 | 0.53 | 1.61 | 4.25 | 811.30 | 2699.11 |
|  | Rivers/Streams | WSA 04 | 75 | 2.20 | 1.23 | 9.42 | 34.40 | 2.48 | 8.15 | 130.25 | 366.19 |
|  |  | NRSA 08 | 19 | 2.07 | 1.18 | 0.68 | 1.22 | 0.91 | 1.38 | 119.80 | 196.40 |
|  |  | NRSA 13 | 31 | 2.48 | 1.92 | 0.98 | 2.38 | 1.33 | 2.61 | 197.55 | 537.53 |
|  |  | NRSA 18 | 29 | 2.10 | 1.59 | 0.63 | 1.34 | 0.78 | 1.28 | 92.75 | 216.71 |
| 12 – Southern Semi-Arid Highlands | Lakes | NLA 07 | 1 | 10.24 | NA | 1.22 | NA | 2.23 | NA | 36.00 | NA |
|  |  | NLA 12 | 1 | 5.31 | NA | 0.00 | NA | 0.57 | NA | 38.00 | NA |
|  |  | NLA 17 | 1 | 12.34 | NA | 0.00 | NA | 0.83 | NA | 13.61 | NA |
|  | Rivers/Streams | WSA 04 | 3 | 1.00 | 0.05 | 1.11 | 1.80 | 0.40 | 0.46 | 41.67 | 34.03 |
|  |  | NRSA 08 | 8 | 1.87 | 1.69 | 0.93 | 1.43 | 1.28 | 1.79 | 570.50 | 1392.19 |
|  |  | NRSA 13 | 4 | 1.97 | 1.00 | 0.02 | 0.05 | 0.31 | 0.30 | 109.00 | 54.73 |
|  |  | NRSA 18 | 2 | 1.35 | 0.62 | 0.25 | 0.35 | 0.37 | 0.32 | 59.34 | 33.06 |
| 13 – Temperate Sierras | Lakes | NLA 07 | 16 | 7.33 | 3.40 | 0.03 | 0.06 | 0.64 | 0.44 | 99.75 | 132.02 |
|  |  | NLA 12 | 14 | 6.77 | 4.34 | 0.01 | 0.03 | 0.98 | 1.01 | 209.21 | 250.12 |
|  |  | NLA 17 | 9 | 8.64 | 2.02 | 0.00 | 0.00 | 1.30 | 1.36 | 253.82 | 199.42 |
|  | Rivers/Streams | WSA 04 | 31 | 2.58 | 3.59 | 0.14 | 0.15 | 0.27 | 0.31 | 61.87 | 44.84 |
|  |  | NRSA 08 | 19 | 1.24 | 0.48 | 0.07 | 0.13 | 0.23 | 0.19 | 65.03 | 42.98 |
|  |  | NRSA 13 | 11 | 1.75 | 1.36 | 0.06 | 0.11 | 0.24 | 0.20 | 113.45 | 90.35 |
|  |  | NRSA 18 | 13 | 2.23 | 2.15 | 0.08 | 0.16 | 0.20 | 0.17 | 61.92 | 41.78 |
