# Supplementary material for: Limited progress in nutrient pollution in the U.S. caused by spatially persistent nutrient sources
Source: PLoS One. 2021 Nov 29;16(11):e0258952. doi: 10.1371/journal.pone.0258952 (PMC8629290; doi:10.1371/journal.pone.0258952)
Supplement: S3 Table — (DOCX) [file pone.0258952.s012.docx]

| **Table S3. Paired t-test results (p-values) and percent change from repeat sample sites between surveys.** | | | | | | | | | | |
| --- | --- | --- | --- | --- | --- | --- | --- | --- | --- | --- |
|  | | | DOC | | NO_3_^-^ | | TN | | TP | |
| Ecoregion | Waterbody | Surveys | % change | p-value | % change | p-value | % change | p-value | % change | p-value |
| All | Lakes | 1 & 2 | -2.94 |  | -52.51 | *** | 0.43 |  | 57.32 | *** |
|  |  | 2 & 3 | 6.60 |  | -35.62 | *** | 9.44 | *** | -17.38 | *** |
|  | Rivers/Streams | 1 & 2 | 40.33 |  | -53.45 | *** | 60.37 |  | 156.36 | *** |
|  |  | 2 & 3 | 4.52 | *** | 16.35 | ** | 1.61 | *** | 17.42 | *** |
|  |  | 3 & 4 | -3.83 | *** | 60.80 | * | -9.10 | *** | -61.59 | *** |
| 5 | Lakes | 1 & 2 | 8.95 |  | -34.81 |  | 14.04 | * | 128.14 | *** |
|  |  | 2 & 3 | 12.95 |  | -68.96 | *** | 1.22 |  | -17.95 | *** |
|  | Rivers/Streams | 1 & 2 | 1.69 |  | -81.46 | *** | 4.90 |  | 64.95 | * |
|  |  | 2 & 3 | -3.61 |  | 53.67 | *** | 1.55 | ** | 52.96 | *** |
|  |  | 3 & 4 | -9.44 | *** | -5.10 | * | -16.32 | *** | -64.28 | *** |
| 6 | Lakes | 1 & 2 | 0.75 |  | -59.59 | *** | 6.94 |  | 186.62 | *** |
|  |  | 2 & 3 | -0.74 |  | -33.77 | * | 22.14 | * | -35.67 | *** |
|  | Rivers/Streams | 1 & 2 | 7.67 |  | -73.70 |  | -21.27 |  | 192.22 | * |
|  |  | 2 & 3 | 3.21 |  | 23.91 | * | 22.53 | ** | 25.42 | *** |
|  |  | 3 & 4 | -3.99 |  | 24.53 |  | 6.48 |  | -48.83 |  |
| 7 | Lakes | 1 & 2 | -20.42 |  | -23.09 | * | -3.02 |  | 156.37 | ** |
|  |  | 2 & 3 | 43.86 |  | -72.11 | ** | 30.01 |  | -6.58 | * |
|  | Rivers/Streams | 1 & 2 | -28.48 |  | -89.40 | *** | -16.21 |  | 158.46 |  |
|  |  | 2 & 3 | 6.77 |  | 4.74 | * | -20.61 |  | -24.52 |  |
|  |  | 3 & 4 | -7.17 | ** | 7.08 |  | 18.35 | * | -25.01 | *** |
| 8 | Lakes | 1 & 2 | 2.57 |  | -46.07 | *** | 9.58 |  | 57.57 | *** |
|  |  | 2 & 3 | 3.21 |  | -30.51 |  | 7.88 | *** | -10.12 | *** |
|  | Rivers/Streams | 1 & 2 | 20.32 |  | -80.57 | *** | 19.82 |  | 120.37 | *** |
|  |  | 2 & 3 | 9.67 |  | 42.44 | *** | 12.63 |  | 14.10 | ** |
|  |  | 3 & 4 | -11.16 | *** | 22.97 |  | -14.97 | *** | -65.22 | *** |
| 9 | Lakes | 1 & 2 | -0.26 |  | -62.94 | *** | 3.76 | * | 20.26 | ** |
|  |  | 2 & 3 | 1.31 |  | -30.96 | * | -0.38 |  | -16.90 | *** |
|  | Rivers/Streams | 1 & 2 | -16.82 | * | -73.98 | ** | -29.90 |  | 4.54 |  |
|  |  | 2 & 3 | 14.06 | *** | -4.76 |  | 9.31 | * | 21.52 | *** |
|  |  | 3 & 4 | -4.34 |  | 185.46 | ** | -11.57 | *** | -59.32 | *** |
| 10 | Lakes | 1 & 2 | -9.52 |  | -60.28 | * | -11.39 | ** | 78.67 | * |
|  |  | 2 & 3 | 8.94 |  | 35.76 | * | 9.65 |  | -41.31 | ** |
|  | Rivers/Streams | 1 & 2 | 25.33 |  | -76.39 | * | 5.55 |  | 106.89 | ** |
|  |  | 2 & 3 | 5.22 |  | 38.00 |  | 7.47 | * | 61.99 | ** |
|  |  | 3 & 4 | 5.29 |  | 32.79 |  | -22.82 | ** | -72.62 | ** |
| 11 | Lakes | 1 & 2 | 17.61 |  | 24.60 |  | -4.80 |  | -4.95 |  |
|  |  | 2 & 3 | 14.82 |  | -48.30 |  | 78.11 |  | 195.82 |  |
|  | Rivers/Streams | 1 & 2 | -2.30 |  | -64.28 |  | -5.56 |  | 119.33 |  |
|  |  | 2 & 3 | 8.77 |  | 108.68 |  | 4.91 |  | 0.85 |  |
|  |  | 3 & 4 | -11.99 |  | -27.69 |  | -15.13 |  | -21.82 |  |
| 13 | Lakes | 1 & 2 | -15.72 |  | -63.56 | * | 19.21 |  | 139.28 |  |
|  |  | 2 & 3 | 46.77 |  | -44.04 |  | 27.89 |  | 19.81 |  |
|  | Rivers/Streams | 1 & 2 | -34.08 |  | -68.76 |  | -7.50 |  | -9.46 |  |
|  |  | 2 & 3 | 22.99 |  | 87.00 |  | 3.58 |  | 85.01 |  |
|  |  | 3 & 4 | 15.11 |  | -27.06 |  | -14.44 |  | -36.86 | * |
| Note: | * | 0.05 > p > 0.005 | | | | | | | | |
|  | ** | 0.005 > p > 0.0005 | | | | | | | | |
|  | *** | p < 0.0005 | | | | | | | | |
