# Supplementary material for: Limited progress in nutrient pollution in the U.S. caused by spatially persistent nutrient sources
Source: PLoS One. 2021 Nov 29;16(11):e0258952. doi: 10.1371/journal.pone.0258952 (PMC8629290; doi:10.1371/journal.pone.0258952)
Supplement: Attachment — Submitted filename: Limited progress in nutrient pollution_Review.docx [file pone.0258952.s013.docx]

**Limited progress in nutrient pollution in the U.S caused by persistent nutrient sources**

Overall, the manuscript is very well written with excellent figures. I would suggest some minor changes in the MS before it can be accepted for publication.

Comments on the title:

The title is a little confusing. Suggest making it clearer.

Do you mean the pollution in the US has not progressed? Or do you mean that the measures of pollution reduction have not been successful? What are you calling “persistent sources” in the title? The term persistent source is too generic, I suggest in being more specific about sources. The title should also state somewhere that you did not collect samples or analyzed the samples but your results are a result of machine learning analyses or modelling. In current state, the title is a little misleading.

Comments on the abstract:

It would be useful to make a brief statement in the abstract about how this study is similar/different from previous studies conducted using machine learning models particularly the one that is referred in this MS the most often (reference # 28). Also, why did you choose year 2000 to 2012 for the analyses?

General comments on the MS

Make sure you are consistent with the use of NO_3_^-^ and nitrate. I suggest spell it at first use and then use NO_3_^-^ consistently through the MS. Also spell other abbreviations once like DOC etc.

You should also state somewhere in the MS what the biases of the models you are utilizing here?

Line 60 and Line 69: Should the reference be written as Abbott et al (year) or Abbott et al (28)? Please check the journal format and make the change accordingly.

Stay consistent with use of either Fig. or Fig or Figure. Choose one and stay with it.

Why are figure legends provided in the middle of the MS. They should either go with the figures or at the end of the MS, all on one page. I hope this gets fixed during publication.

Figure 1: The asterisk used to represent significant increase and significant decrease are confusing. Does asterisk represent difference for all years in one specific region? Please clarify.

Page 9: Please give some introduction on Spearman rank correlations

Line 208-210: Explain why region 12 was not used in Figure 1? Region 15 is not even labeled in Figure 1

In general, it would be useful to provide little more introduction about models used in the MS and their significance.

Line 286: Describe what Lentic and Lotic systems are?

Fig 4 (a) colors are too close to each other so it is hard to find different regions on it.

Line 507: Has the dual approach that you used here been used before? Line 506-510 : It would be useful to describe your approach in the abstract as well

Line 529-539: are these the conclusions of this study? If yes, it would be useful to label them as conclusions.
