# Supplementary material for: Limited progress in nutrient pollution in the U.S. caused by spatially persistent nutrient sources
Source: PLoS One. 2021 Nov 29;16(11):e0258952. doi: 10.1371/journal.pone.0258952 (PMC8629290; doi:10.1371/journal.pone.0258952)
Supplement: Attachment — Submitted filename: Frei_ResponsetoReviewers.docx [file pone.0258952.s014.docx]

**Comments to the Author**

**Reviewer #1:** I was really excited to read this paper about the persistence of nutrient loading across the United States. The paper introduction raises the interesting question of variation in nutrient persistence pathways: how surface water responds to reductions in nutrient inputs might follow various pathways depending on X, Y, Z. Unfortunately, the authors didn’t address this exciting question with their analyses – instead the analysis focused on understanding what land use was associated with concentrations of N, P and DOC. I think there is a lot of potential here, but I struggled with the disconnect between the gap articulated in the introduction and the analyses. I think there are two pathways forward: 1) keep the analysis as is. The gap could then be that there have been analyses of controls on nutrients, but they could be affected by temporal variation among sampling dates. This analysis tests that assumption – for the most part spatial patterns are persistent, so we can really trust the machine learning analysis. I think this would be a less interesting paper. We already know that land use affects nutrient concentrations, that these affects differ among nutrients. I wasn’t really clear what was novel about that part of the analysis. 2) What I think would be a more novel contribution would be to explore different nutrient trajectories, and where those are happening. I could imagine a similar ML approach, but using changes in nutrient concentrations (or changes in rank within a watershed?) as the response variable. Would allow you to address – what are the different pathways? What land uses are associated with increases/decreases? Are there differences among watersheds (or regions, but see my comments on that below) that are related to regional/local nutrient management approaches? For example, it would be interesting to see if agriculture is associated with increases in some regions and decreases in others (or no change in others) and if that were linked to differences in policies, etc. I recognize that this would be a substantially different paper, but I think the questions raised in the introduction are exciting and addressable using this dataset with some modified analyses.

We are very grateful for these insightful and creative ideas. We see how there was a disconnect between the framing of the introduction and the analyses that we performed. Since our initial submission, additional chemistry data has been released by the EPA. This has allowed us to extend the analysis through time, giving us much greater confidence in trends in nutrient and carbon concentration and leverage. However, the associated catchment characteristics (including land use) are not yet available. Consequently, we were unable to perform the proposed analysis comparing land use with degree of change. We plan on doing this analysis once the data are available, and we would be happy to collaborate with the reviewer for a future project if they are interested.

In response to these critiques, we have realigned the introduction and hypotheses to emphasize that the focus of the paper is on trends in nutrient concentrations for different ecoregions. While we do not yet have adequate data to establish what those trends are correlated with, we emphasize three main contributions of this paper:

1. A large-scale and relatively high spatial resolution analysis of temporal change in nutrients and carbon (leverage and concentration)
2. An exploration of the ecohydrological patterns within that dataset (particularly spatial persistence and patch size), which allows the framing of hypotheses for why concentrations are or aren’t changing
3. A large-scale testing of relationships between catchment characteristics and nutrient concentrations

We recognize that contribution 3 could be considered less novel, but we believe it is still worth publishing for two reasons. First, it tests how general are observed relationships between land use and nutrient concentrations. These relationships are often observed at smaller spatial scales or with less spatial resolution. Second, this analysis tests how diverse are the outputs of multiple machine learning algorithms. This has certainly been done “under the hood” in different studies, but this is one of the first water chemistry studies that provides clear visualizations of various methods.

Some more major comments about the analysis approach:

1. Spatial persistence
a. I struggled with the various ways that spatial persistence could be interpreted. If patterns are the same between sampling points, then that seems straightforward - the patterns are the same, there hasn't been a change. But if the patterns are different, how do you know if that was because the sampling events were not representative of the first time period or because there was a change in the spatial patterns of inputs (or sinks). If you expect differences in responsiveness to point vs diffuse inputs of nutrients, then it would make sense that spatial patterns would change (assume that point sources and diffuse inputs are all reduced at the same rate [this is unrealistic, but a useful thought exercise], areas most affected by point sources might experience a greater decrease in concentration compared to those most affected by diffuse sources). You are trying to use one analysis to assess both spatial and temporal patterns, but I don’t think you can use this analysis to say whether or not snapshot surveys are representative of temporal trends (your question 2).

The reviewer correctly identifies one of the weaknesses of the spatial persistence metric: it does not, on its own, indicate reasons for temporal change in spatial pattern. Quantifying spatial persistence can indicate potential drivers of change, but it cannot establish them on its own. As mentioned in the last response, we plan on coupling large-scale nutrient inventory data and land use data with nutrient concentrations and fluxes in a future analysis, which will hopefully reveal the proximal causes for changes in persistence. We have updated the text throughout to make the limitations of this approach clearer. By integrating the additional sampling dates, we can be surer of the temporal trends while using the spatial persistence metric as intended to assess how representative of relative nutrient status a single sampling is.

b. A second question about the use of these stems from the statement in the text that: “if solutes have low spatial persistence (perhaps due to asynchronous variation) then analyses of patch size and nutrient contribution made for a single moment in time are not reliable indicators of long-term nutrient dynamics.” If low spatial persistence means that you cannot assess things like patch size, were regions/analytes with low persistence excluded from patch size and leverage analyses? Or, would it be interesting to see how patch size and leverage change for those between the two sampling times?

We appreciate these insightful comments and critiques. We apologize for the lack of clarity in the original phrasing. Even in a system with low spatial persistence, you can still assess the short-term patch size and leverage. It is only the longer-term dynamics that remain out of reach in a low persistence system. As the reviewer points out, changes in patch size and leverage are of great interest, and indeed, based on previous work [1,2], we expect both of these parameters to change in response to hydrological variability, terrestrial and aquatic nutrient demand, and temporal changes in nutrient sources. Consequently, regions and analytes with low spatial persistence were not excluded from the patch size and leverage analyses.

2. Use of ecoregions
a. These ecoregions are really large and encompass a wide range of ecosystems, climate, nutrient management strategies, nutrient management challenges. Some more focused discussion about how you expected patterns to vary among ecoregions based on their hydrology, climate, vegetation, or land use would help justify the use of ecoregions and interpret differences among ecoregions. As is, there are interesting differences among regions, but the reasons for these differences are not explore.

Thanks for this feedback. We now provide more justification for why we used ecoregions. Specifically, we do not hypothesize that they are perfect ecohydrological delineations. Instead, we use them as convenience boundaries between areas with broadly different characteristics. This is in line with their original intent [3]. Splitting the analysis in this way allows us to assess ecohydrological patterns in various socioecological contexts. This has multiple benefits, for example, it demonstrates where infrequent synoptic sampling is more or less representative of long-term conditions. It also corresponds with spatial scales of management by connecting the county and state scale with regional scales.

b. I was very confused about the estimates of leverage using the ecoregions. These are not watersheds, so it doesn’t make sense how or why you would calculate leverage for all the catchments within a region relative to the largest catchment in the region. For the temperate eastern forests, the ecoregion “outlet” would be the Mississippi River, but many (most?) of the “subcatchments” are not within the Mississippi River basin, and many of the subcatchments of the Mississippi River basin are not within that ecoregion. It seems to me that using watershed boundaries rather than ecoregions would make much more sense for these analyses which are designed for making comparisons within watersheds.

We considered multiple ways of calculating the leverage metric, including with topographic watersheds as proposed by the reviewer. Ultimately, we opted to use ecoregions for a few reasons. First, even though the samplings included thousands of locations, there were usually only a few points within individual watersheds, except for large rivers such as the Mississippi and Colorado. Second, many of the sampled locations were not nested within larger watersheds, such as those draining directly to the ocean or occurring within endorheic (terminal) basins. Small watersheds are influential in determining lateral nutrient transport as well as being indicators of local nutrient management and conditions (42,55,68). Third, splitting by ecoregion allows grouping of sampling points with more similar land uses and climatic conditions than would be seen within a single large watershed. This allows for more sensitive comparison of nutrient source and sink dynamics either in the terrestrial environment or in the aquatic network. For example, two sampling locations that are adjacent spatially but occur on different branches of different watersheds may have more in common than nested watersheds that are more geographically separated.

As stated by the reviewer, the outlet of the largest watershed within the region becomes the relative zero point for both upstream points and unnested points. This infringes the assumption of transfer of mass through the outlet point, but because we calculate leverage as a percent, it is still interpretable for nearby, unnested sites. For the Mississippi, we compared the results when calculated as a watershed, and found nearly identical patterns, except it excluded the coastal and adjacent unnested watersheds. We now provide a more complete justification for this choice.

3. Mass balance approach
a. This really relies on specific discharge being consistent within a watershed or region, which is not the case (as your Figure S4 illustrates well, especially for regions 5,6,7 and 8 in which specific discharge varies by what looks like an order of magnitude).

We agree with the reviewer that this is a source of uncertainty in our estimates. However, the choice of using ecoregions reduces the order of magnitude variation observed across all sites to a much more acceptable level (e.g., ecoregion 7 showed the greatest variability with an interquartile range that varied by 1.7 times). The alternative of directly comparing yields drastically reduces the number of locations included in the analysis to sites with a gauge. This adds another type of uncertainty to the analysis, which we believe is more systematic and problematic. We now explore these tradeoffs in more detail in the methods section.

Specific line comments:

Line 32: how are you defining nutrient resilience? I'm not sure how you reach this conclusion from the analyses

We removed the mention of nutrient resilience and further clarified our position.

Line 53: Spatiotemporal patterns of what?

Added “spatiotemporal patterns of nutrient concentrations”.

Lines 54-57: Can you say more about these trajectories - what are they? Is the point here to refute the legacy hypoth? Or just say that there are more patterns?

We have added detail about the heterogeneity of the patterns and specify how they relate to our hypotheses. We try to strike a balance between describing the overall patterns of interest and diving too deep into local variation.

Lines 57-59: unclear here exactly what is and isn't known, what the gap is, besides a need for expanded analysis. What will the expanded analysis tell us?

We use this nationwide analysis to expand the analysis to move beyond one watershed or region to the entire contiguous U.S. We propose that this has merit on its own, and as stated in the text, we use the expanded analysis to assess effectiveness of past policy and inform future interventions.

Line 64: unclear what the correlations are between - the locations at diff times?

Yes. We updated the text to clarify this point.

Line 69: Can you define leverage here?

Added a sentence to quickly define the term.

Lines 77-79: I don’t think you can really address Q2 since you aren't comparing the snapshots with more temporally resolved data

We have revised Q2 to address spatiotemporal patterns more generally, which is what we actually quantified. To the reviewer’s point, however, while we don’t have more temporally resolved data, the fact that rank correlations were close to 1 for most solutes implies that nutrients were spatially persistent through time, which is a good sign that the EPA surveys are effective at capturing temporal trends. It is unlikely (though not impossible) that there would be persistence this high if the snapshots were not capturing the spatiotemporal variability (e.g., see [1]).

Line 80 (Q4): ok - but you focus your results on the concentration results, not the leverage results. If the leverage results are what are most important, then it might make more sense to put those in the paper and put the concentration results in the supplemental

Leverage (associated with flux) and concentration (associated with local conditions) are complementary metrics. Flux is more associated with coastal eutrophication while concentration is more associated with ecological status of freshwater bodies (e.g., algae and other organisms experience concentrations, not fluxes). Because the leverage results have the added sources of uncertainty mentioned above (i.e., specific discharge variability and watershed-ecoregion mismatches), we concentrate on the concentrations for this part of the analysis.

Lines 113-117: how would other results change if you restricted the 2008 study to include the same range of catchment sizes?

Our temporal change analysis uses only the sites that were repeated across samplings. Thus, we are only comparing the same range of catchment sizes from the WSA 2004 survey to the NRSA 2008 survey. This provides the most statistical sensitivity and eliminates any uncertainty associated with changes in sites across dates.

Line 129: boxplot is a visualization tool, not a hypothesis test, but more importantly, unclear what is being compared to what in these boxplots and t-tests

We updated the text to specify that the boxplots and t-tests are assessing changes in nutrient concentrations from survey to survey (i.e., across dates).

Line 131: is there evidence that nutrient management would vary across these regions?
Do you have specific hypotheses about how patterns of nutrient persistence might vary across the regions? I understand that they are different, but how do you expect those differences to manifest in the response variables that you are focusing on? I'd be concerned that the differences are by chance, rather than because of something relevant to the ecoregion or how the ecoregion was designated.

There is evidence that nutrient management varies across these regions [4,5]. However, this study is meant as a survey of ecohydrological patterns and a test of changes through time. After discussion as a group, we feel it is beyond the scope of this study to develop detailed hypotheses for each of the ecoregions. In this revision, we try to strike a balance between detail and general interest. We direct readers to new nationwide nutrient inventories and will follow up with future work after catchment characteristics are available across sampling dates.

Line 170-171: Does this mean that other analyses only valid for areas with spatial persistence?

As discussed above, this means that there is greater uncertainty surrounding the patch size and leverage analyses if spatial persistence is low.

Line 181: a table with the different parameters would be really helpful – how they are calculated, inferences

Thank you for this suggestion. In lieu of a table, we have substantially strengthened our description of the methods in the manuscript.

Line 186-187: What was analyzed for these parameters?

Clarified by specifying that we use concentration data for these analyses.

Line 197: are these done for all samples/sites? Or just where there was spatial persistence? Was it done for a single sampling point (early/later?) or all data together?

Yes, these analyses use all the data across surveys and ecoregions regardless of spatial persistence. We further delve into the low persistence issue in the discussion.

Line 226: how are you calculating leverage if not all the subwatersheds are within the largest watershed within each region?

See response on page 4. We have updated the description in the methods.

Lines 227-229: you found similar specific discharge between dates, but not within ecoregions, which was the assumption that you were testing.

Most of the sites within each ecoregion show a relatively narrow range of specific discharge, but we now discuss those that infringe the assumption.

Lines 241-243: these interpretations are based on assumption of consistent within-region specific discharge. How might violations of that assumption (documented in S4, but maybe worth pulling the data that are associated with Q data and seeing how sensitive your results are to this assumption?) affect your interpretations?

We now discuss this point, specifically how systematic differences in specific discharge could influence our estimates of uptake and production.

Line 252: how were these models selected? What are the important differences among them?

The models were selected based on availability, which is correlated with how much they are used. The goal of the approach was to use as many model types as possible so that the analysis would not be reliant on a single algorithm. If there had been more available in the SciKit Learn library in Python, then we would have used them. From lowest to highest complexity, models go from decision tree regressors, to random forests, to gradient boosting regressors, to multilayer perceptrons. Decision tree regressors are basically a single tree structure that in a pair-wise way reduces input features into a single output prediction. They tend to have high bias and low variance. Seeking to improve on those limitations, random forests make predictions that are the average from many decision trees working independently. And gradient boosting regressors are essentially random forests that are built in a less random way. As each new "tree" is added to the forest, it is added according to the "gradient of a loss function," which allows each new tree to target specific weaknesses in the forest's overall behavior. In theory gradient boosting regressors should usually do better than other statistical machine learning models, but this is not always the case. And in many industrial ML applications, people apply a suite of models and just pick the one that happens to work best on that dataset (e.g. https://www.datarobot.com/). Finally, multilayer perceptrons are built around a totally different concept where artificial neurons compute predictions from input data in an integrated network similar to that of the human brain. Despite these fairly significant differences, we were impressed by how the model's behaviors were more similar than not in almost every situation. And again, the point was that we wanted another metric to gauge how well we could trust the models. We were operating under the assumption that multiple types of models suggesting the same kinds of relationships constituted strong evidence that those relationships reflect reality. We have expanded the description of how models were selected in the methods.

Line 268: what data were missing? Wasn't this dataset based on the water quality data? How many data points were removed for missing values?

In both stream and lake data, the dropped values were missing at least one water chemistry data parameter. None of the input features (values used to make the predictions) were missing for any samples. For the lake data, 20 out of 4,716 data points, or 0.4% of the data, were excluded because of missing chemistry data. For the stream data, 59 out of 5,758 data points, or 1% of the data, were excluded because of missing chemistry data.

Line 285: really over the two 5-year study periods, since you only compared changes over 5 years.

We extended the analysis to include stream data until 2019 and lake data until 2017. We updated the manuscript to reflect these time periods.

Lines 285-289: reminder needed here - are these based on the paired sites only?

Yes, thank you.

Line 315-316: curious about how you interpret the differences in patch-size between continental and regional analyses - patch is scale-dependent (this isn't really surprising), but what does it mean for nutrient management?

We agree with the reviewer that the scale-dependence is not surprising, as this analysis is sensitive to sample size. The scale-dependence could emerge from several sources, but it may simply be a consequence of resolution. We develop this a bit more in the manuscript, mainly emphasizing that the specific cutoffs are less useful than the overall pattern of relatively small watersheds acting as critical source areas.

Lines 335-336: shouldn't this be stated, “most catchments had low leverage”?

Yes, thank you.

Line 341: dilution doesn't change the mass balance. I suspect that some of these extreme outliers are due to violations of the assumption that specific discharge is the same within ecoregions.

Corrected, thank you. We discuss the limitations further in the manuscript.

Line 403: has MAAT been defined? If so, it's been a while and worth spelling out here.

Added.

Line 426: how do we know persistence is due to diffuse sources and not point sources (or both)? If concentrations are increasing or staying the same, that might indicate no progress on either front.

Because point sources are regulated more rigorously and are easier to address managerially, they have decreased more systematically than nonpoint sources (e.g., [6]). Thus, we attribute the persistence to continued nonpoint loading. Clarified in text.

Line 431: drivers of nutrient concentrations

Changed.

Line 435: this seems like a distinct point from the first one, but I'm not sure I understand it.

Revised for clarity.

Lines 445: Or....5 years is not a very long time to measure the impacts of nutrient management. I would be really interested to see an analysis that focuses in on the outliers here. I know the persistence analysis is for the whole dataset (or by region), but could you pull out those sites that changed a lot in rank (or even just look at change in rank as a response variable?) to understand why nutrients persist in some places and not in others. The threshold for persistence seems liberal in my view - at the threshold there is still a lot of change! At any rate, I'm confused about the interpretation of the persistence story, if it's the same, then there hasn't been a change, and you can do all the other analyses, if not then you can say that these are real spatial patterns and do the land use analysis. But I don't think you can use this (esp with a 5 year period) to say that we're not making progress with nutrient management. It's not a long time period and the analysis approach can be interpreted in importantly different ways.

Thank you for this criticism. With the expanded datasets, we are more confident that nutrient concentrations are not improving, and that spatial persistence helps explain why to some extent.

Lines 455-456: also to me means that ALL sources of nutrients have persisted, and that there isn't a difference in the persistence of point and diffuse sources over this time period

Revised.

Lines 460-462: BUT - this also only captures spatial patterns of nutrient inputs during baseflow. Seasonal high flows or storm events may activate different sources that may or may not be spatially correlated with baseflow sources and may nore may not be spatially persistent.

See responses on page 5 and earlier. While this experimental design does not capture high flow events, previous work suggests the overall pattern is conserved across the hydrograph [1,2,7]. The fact that these surveys all capture baseflow conditions from the early 2000s to 2017-2019 increases our confidence in the data and our interpretations.

Lines 479: but differences in patch size between regional and continental scale analyses suggests that patch size varies with scale of analysis. If you had access to data for smaller catchments, you might find yet another answer.

While the spatial resolution (points per area) directly influence the ability to detect changes in variance, the extent of the sampling does not necessarily do so. For example, it is likely that with the continental scale analysis, we are detecting the ecoregion collapse points that occur at larger scales. We try to more directly discuss the limitations and implications here and throughout the paper.

Line 511: Can you define scale of headwaters again here?

Clarified.

Lines 517 onward: Can you link these implications specifically to the model results?

We have revised to make it clear what results (both from this and other studies) are associated with what interpretations.

Lines 532-533: spatial patterns were persistent, but the increases in concentrations is relevant too!

Agreed. Added.

Lines 534: can you highlight some of these? Is this new? Comparisons with other analyses?

Expanded.

Fig 2. Is the persistence value for NO3 in region 13 missing? X-axis label missing for panel a

Fixed. Thank you for pointing out these issues.

Fig 4a. these are really hard to see because the axes are such large ranges and most of the values are small

We simplified the colors on Fig 4A to show general leverage patterns across catchment sizes.

Fig 4b. The removal/production don't seem quite right here since you are assuming constant specific discharge. I see the value of making the simplifying assumption, but it does challenge this interpretation

The mass balance interpretation is based on average leverage values which would be less affected by specific discharge than individual leverage values. We have tried to better integrate this caveat throughout the manuscript.

Fig 6 scatterplots: maybe because of the transparency - the wetland points look like the urban color, and other shades are also inaccurate between plot and legend.

We fixed the color discrepancy.

how did the year of sampling play out in these models? Would be really neat if you could see how the models vary from year to year

We agree with the reviewer, and plan to do this for all the samplings once the catchment characteristics are available at a national scale. We are particularly interested in testing the nutrient legacy hypothesis more directly by comparing past land use with current chemistry. For the data we have in hand, Fig. 6 suggests that sample year was not an important predictor of nutrient concentration (i.e., the same relationships persisted across the samplings).

**Reviewer #2:** Line 86. Is a five year period really adequate to determine temporal trends. Please justify.

We are grateful for this criticism and the careful read of the reviewer. In this revision, we have included newly released EPA data that extends the rivers and streams data to 20 years and the lake data to 10 years.

Line 398-399. SUrprised ag did not have greater impact on nitrate.

As were we. We try to develop this point in the manuscript. Depending on the region, nitrate could be sourced primarily from agriculture, wastewater, urban runoff, or atmospheric deposition.

Line 436-438. WHere is the analysis on what the evolution of ag practices was during your time frame? Many areas would have had very little change during your study period. To call out the potential role of ag management changes during this period need to have more information on this.

We agree with the reviewer that a detailed time series analysis of the land use over these samplings would be valuable. See our responses to Reviewer #1 on this topic. For the purposes of this manuscript, analyzing the evolution of agricultural practices is beyond the scope of this paper, but we point readers toward relevant literature.

Line 526-528. There is little on fertilizer use especially distribution of use and rates. Might be worth calling out there is a need in this area.

Our data do not speak to this directly, but we have added a mention.

**Reviewer #3:** Some of the supplementary figures embedded in the MS are in EPS format which was inaccessible from my device. Make sure to save it in correct format and check its accessibility. Specifically, check the format of the figures S1 through S10.

We apologize for the inconvenience. Our instructions were to submit figures in EPS format, and we will double-check to make sure they are formatted correctly in the next iteration.

**Attached Word Document:**

Overall, the manuscript is very well written with excellent figures. I would suggest some minor changes

in the MS before it can be accepted for publication.

**Comments on the title:**

The title is a little confusing. Suggest making it clearer.

Do you mean the pollution in the US has not progressed? Or do you mean that the measures of

pollution reduction have not been successful? What are you calling “persistent sources” in the title? The

term persistent source is too generic, I suggest in being more specific about sources. The title should

also state somewhere that you did not collect samples or analyzed the samples but your results are a

result of machine learning analyses or modelling. In current state, the title is a little misleading.

We appreciate the input from the reviewer and the careful read of our manuscript. We recognize that the title is somewhat generic. Yet, after discussion and trying several alternatives, we found that it was more difficult to interpret the main message. While “progress in nutrient pollution” is technically ambiguous, we have received feedback that readers interpret it correctly (i.e., it means there has not been much decrease in pollution). Likewise, it is specified in the first lines of the abstract that this is an analysis of EPA data rather than sampling that we undertook personally. We are happy to revisit if the reviewer and editor still find the title unacceptable.

**Comments on the abstract:**

It would be useful to make a brief statement in the abstract about how this study is similar/different

from previous studies conducted using machine learning models particularly the one that is referred in

this MS the most often (reference # 28). Also, why did you choose year 2000 to 2012 for the analyses?

This manuscript uses some of the ecohydrological metrics in reference 28, but otherwise has very little in common with it. For example, that study did not use any machine learning models and was constrained to a very small spatial scale. Additionally, that study did not interpret the distribution of leverage values to infer uptake or release.

We initially analyzed data from the 2000-2012 time-period because that was the data available from the EPA. Thankfully, further data has been released and we have extended our analysis to include the most recent lake and river/stream data, which extends our study’s temporal extent to 2019.

**General comments on the MS**

Make sure you are consistent with the use of NO 3 - and nitrate. I suggest spell it at first use and then use NO 3 - consistently through the MS. Also spell other abbreviations once like DOC etc.

Thank you, we have updated this.

You should also state somewhere in the MS what the biases of the models you are utilizing here?

We have updated the methods section describing the models, including a discussion of limitations and biases.

Line 60 and Line 69: Should the reference be written as Abbott et al (year) or Abbott et al (28)? Please

check the journal format and make the change accordingly.

Corrected, sorry for the inconsistency.

Stay consistent with use of either Fig. or Fig or Figure. Choose one and stay with it.

Yes, thank you for pointing this out.

Why are figure legends provided in the middle of the MS. They should either go with the figures or at

the end of the MS, all on one page. I hope this gets fixed during publication.

This format was requested by the journal for the initial submission.

Figure 1: The asterisk used to represent significant increase and significant decrease are confusing. Does

asterisk represent difference for all years in one specific region? Please clarify.

We compare changes in concentration using paired t-tests. Most sites were sampled only once per survey but some were sampled repeatedly. In this case we took the mean of the samples (per survey) for the t-tests. Therefore, the asterisks indicate significant increases or decreases between paired sites between surveys. We have updated this in the methods.

Page 9: Please give some introduction on Spearman rank correlations

Added.

Line 208-210: Explain why region 12 was not used in Figure 1? Region 15 is not even labeled in Figure 1

Ecoregion 12 had only 6 locations, and ecoregion 15 had no sampling locations. We now specify that we include only ecoregions with adequate sampling points.

In general, it would be useful to provide little more introduction about models used in the MS and their

Significance.

We have expanded the description of the models, explaining that our goal was to test all available machine learning models. Because these methods are relatively new to ecology, we wanted to provide a fair and comprehensive comparison of their performance.

Line 286: Describe what Lentic and Lotic systems are?

Added.

Fig 4 (a) colors are too close to each other so it is hard to find different regions on it.

We changed the colors in the figure to make it more legible.

Line 507: Has the dual approach that you used here been used before? Line 506-510 : It would be useful

to describe your approach in the abstract as well

This “dual approach” has surely been proposed elsewhere, but we now emphasize it further as suggested.

Line 529-539: are these the conclusions of this study? If yes, it would be useful to label them as

Conclusions.

We added a conclusions subheading.

References

1.	Abbott BW, Gruau G, Zarnetske JP, Moatar F, Barbe L, Thomas Z, et al. Unexpected spatial stability of water chemistry in headwater stream networks. Grover J, editor. Ecology Letters. 2018;21: 296–308. doi:10.1111/ele.12897

2.	Gu S, Casquin A, Dupas R, Abbott BW, Petitjean P, Durand P, et al. Spatial Persistence of Water Chemistry Patterns Across Flow Conditions in a Mesoscale Agricultural Catchment. Water Resources Research. 2021;57: e2020WR029053. doi:10.1029/2020WR029053

3.	Omernik JM. Ecoregions of the Conterminous United States. Annals of the Association of American Geographers. 1987;77: 118–125. doi:10.1111/j.1467-8306.1987.tb00149.x

4.	Sabo RD, Clark CM, Bash J, Sobota D, Cooter E, Dobrowolski JP, et al. Decadal Shift in Nitrogen Inputs and Fluxes Across the Contiguous United States: 2002–2012. Journal of Geophysical Research: Biogeosciences. 2019;124: 3104–3124. doi:10.1029/2019JG005110

5.	Byrnes DK, Meter KJV, Basu NB. Long-Term Shifts in U.S. Nitrogen Sources and Sinks Revealed by the New TREND-Nitrogen Data Set (1930–2017). Global Biogeochemical Cycles. 2020;34: e2020GB006626. doi:10.1029/2020GB006626

6.	Topp SN, Pavelsky TM, Stanley EH, Yang X, Griffin CG, Ross MRV. Multi-decadal improvement in US Lake water clarity. Environ Res Lett. 2021;16: 055025. doi:10.1088/1748-9326/abf002

7.	Shogren AJ, Zarnetske JP, Abbott BW, Iannucci F, Frei RJ, Griffin NA, et al. Revealing biogeochemical signatures of Arctic landscapes with river chemistry. Sci Rep. 2019;9: 1–11. doi:10.1038/s41598-019-49296-6
