# Supplementary material for: Limited progress in nutrient pollution in the U.S. caused by spatially persistent nutrient sources
Source: PLoS One. 2021 Nov 29;16(11):e0258952. doi: 10.1371/journal.pone.0258952 (PMC8629290; doi:10.1371/journal.pone.0258952)
Supplement: Attachment — Submitted filename: Response to reviews round 2.docx [file pone.0258952.s015.docx]

Our responses are noted below in blue.

**Reviewer comments**

Reviewer #1: The authors have done a thorough revision, and the resulting manuscript is much stronger and clearer. I was also excited to see the inclusion of new data and the longer time period of their analysis. They did a great job revising the introduction and discussion to better match their analyses, and the methods now provide better detail about the many metrics that they use in the paper. The revisions address nearly all of my concerns.

We are glad the reviewer found the updates helpful and important. Their input was key to improving the manuscript.

My one remaining concern is the use of ecoregions as the unit of analysis for the leverage analysis. I would suggest that your rationale for using them include the good reasons that you can't use watersheds - but it doesn't mean that ecoregions are an appropriate substitute. The analysis doesn't make physical sense if the watersheds being compared aren't hydrologically connected. I don't see how you can make any valid inferences from this analysis. I'm also confused by your third point - many of the ecoregions you use are very large and don't have similar land use or climate (e.g., Maine and Florida). While you acknowledge this in the methods, the results and discussion interpret the data as if these were nested watersheds – referring to smaller watersheds as “subwatersheds” and making inferences about nutrient retention between non-nested watersheds. I don’t think the leverage analysis is critical to the story here, and I would suggest cutting it. Otherwise, I think the paper is really nice.

We see the reviewer’s points and have added caveats to avoid overinterpreting the leverage analysis. We have also clarified the confusing wording in the results and discussion and added more detail in the methods, including some of the points the reviewer has listed above concerning the limitations of this method (see lines 276-281).

For the trends, I’m curious whether the authors think these are due to variation in nutrient inputs and retention alone, or how much could be caused by interannual variability in hydrology. This could be a small point for the discussion.

We agree that hydrological interannual variability could affect apparent trends, though there were not substantial differences in specific runoff across dates for the stations with discharge measurements. We added some text to acknowledge this point in lines 543-544.

Reviewer #3: All comments have been addressed

We are grateful for the careful and insightful review process. Thank you!
